# A case report and a call for early recognition: post-operative Pyoderma Gangrenosum after breast-conserving surgery in a locally advanced breast tumor

**DOI:** 10.1080/23320885.2024.2304620

**Published:** 2024-01-18

**Authors:** Abdulrahman Hashim, Fabien Boucher

**Affiliations:** a Department of Plastic and Reconstructive Surgery, Hospices Civils de Lyon, Hôpital de la Croix Rousse, Lyon, France, Department of Surgery, University of Jeddah Medical School, Jeddah, Saudi Arabia; b Department of Plastic and Reconstructive Surgery, Hospices Civils de Lyon, Hôpital de la Croix Rousse, Lyon, France

**Keywords:** Breast reconstruction, breast pyoderma gangrenosum, post-surgical pyoderma gangrenosum, case report

## Abstract

We present a case of a patient addressed to our plastic surgery department after a breast tumor resection and subsequent necrosis and ulceration of the right breast. A diagnosis of pyoderma gangrenosum was made based on the clinical criteria, early recognition resulted in complete recovery without further reconstructive surgery.

## Introduction

Pyoderma gangrenosum (PG) is a rare inflammatory cutaneous disorder manifested by rapidly evolving large, painful, necrotic ulcers with violaceous, undermined edges.

Mostly considered idiopathic but the precise pathogenesis is unclear, neutrophil dysfunction, genetic variations, and systemic autoinflammatory disorder have been associated with the condition [1]. Postoperative breast PG has been reported in the literature either in primary oncological, or secondary reconstructive surgery with autologous tissue or implant-based reconstruction [2–5].

Post-surgical pyoderma gangrenosum (PSPG) is a diagnosis of exclusion but with a course that mimics surgical site infections and in severe cases even necrotizing fasciitis. A process that provokes a diagnostic dilemma.

Due to the absence of pathognomonic histological and laboratory findings; patients often undergo unnecessary treatments and surgical interventions due to misdiagnosis. This delay can lead to aggravation of the ulceration and severe morbidity in involved patients.

## Case

We report a case in line with the SCARE 2023 guidelines [6] of A 68-year-old Caucasian female with known POLYCYTHEMIA VERA and V6l7F JAK 2 mutation, treated with (HYDREA) HYDROXYCARBAMIDE for 13 years with no other comorbidities, allergy, or history of smoking was addressed to our service by a surgical oncologist working in a private institute for the management of an evolving right breast ulcer twelve days after she underwent breast-conserving surgery (BCS), using a direct lateral scar that was associated with a sentinel lymph node biopsy (SLNB) for a locally invasive 10 mm ductal adenocarcinoma.

**Figure 1.**
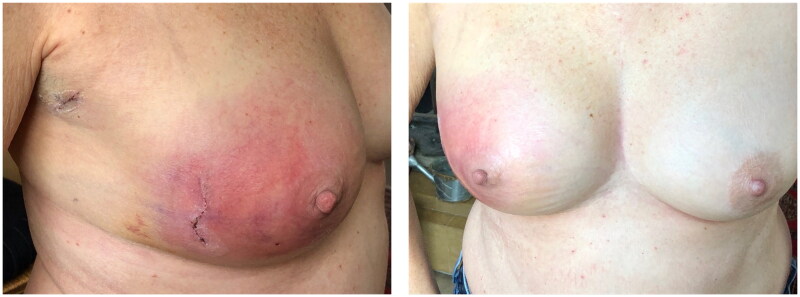
(POD7) Breast erythema that was managed as a simple surgical wound infection.

The primary BCS and SLNB went without any complications, the patient was discharged on the same day before she presented to her surgeon on POD7 with a painful right breast associated with outer quadrant swelling and erythema that was initially treated as a post-surgical wound infection with Pristinamycin orally (Figure 1).

The patient was seen at POD9 in the office and a 5 × 7 cm ulcer was noted at the surgical site with high-grade fever, CRP 165 250 mg/l, and WBC of 13,000/mm^3^, So a decision was made to take the patient to the operating room for surgical debridement, washout in addition to tissue cultures.

The patient was started on intravenous Amoxicillin/clavulanic acid 3 G/24H while awaiting the microbiology laboratory results with no response and persistence of pain and fever, a breast ultrasound was done and 15 mL of serosanguinous fluid was aspirated and sent for further microbiological analysis.

**Figure 2.**
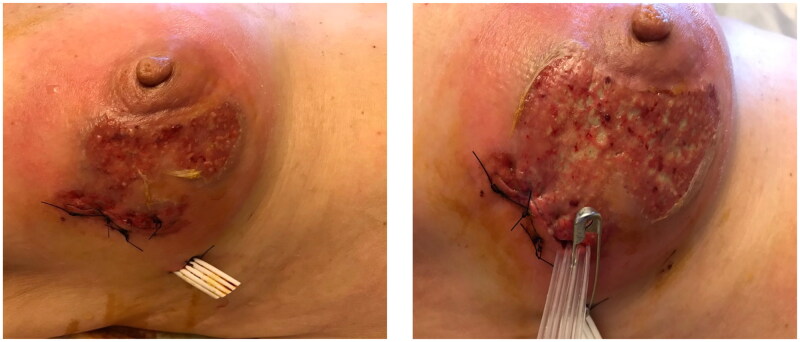
(POD10 and 12) the patient was taken to the or for debridement twice with worsening and rapid progression of the ulceration with each operation (Pathergy).

Two days later, a rapidly evolving inflamed, irregular, and deeply eroding ulcer with a sharply circumscribed border sparing the nipple-areola complex (NAC) was noted at the site of the surgical wound and was associated with severe pain, necrotic hemorrhagic pustules and bullae formation on the outer quadrant of the right breast, the patient was taken for a second look in the or fearing a rapidly evolving necrotizing fasciitis, further debridement, and washout was done (Figure 2).

At POD12 the patient was addressed to our plastic surgery department, on arrival the ulceration had already occupied the majority of the outer quadrant of the breast with multiple zones of necroses (necrosis), erythema of the entire right breast with diffuse tenderness, all the Microbiological workup and blood-cultures were negative at that point. After discussing with our collogues (colleagues) in infectious disease and internal medicine, we decided to start treatment for Pyoderma gangrenosum as the patient fulfilled major and minor diagnostic criteria (Table 1).

**Table 1.** Proposed diagnostic criteria of pyoderma gangrenosum (PG) by Su et al.

Major criteria	Minor criteria
Rapid progression of a painful, necrolytic cutaneous ulcer with an irregular, violaceous, and undermined border. Exclusion of other causes.	Pathergy and worsening of symptoms with minor trauma, or clinical finding of cribriform scarring. Systemic diseases associated with PG. Histopathologic findings (neutrophilic infiltration). Rapid response to systemic steroid treatment.

**Figure 3.**
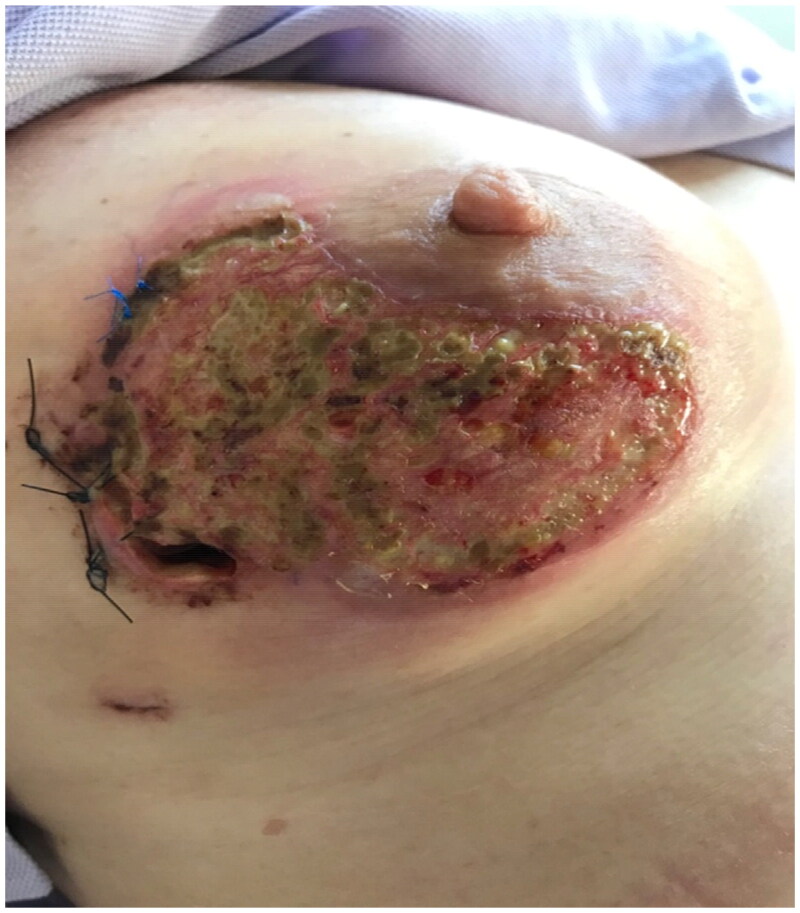
(POD13) 24h after the establishment of the diagnosis of pyoderma gangrenosum and starting steroids, we can see the disappearance of erythema and subsequently pain.

A biopsy was done under local anesthesia in the clinic that eventually showed neutrophilic infiltrate and the patient was admitted for treatment with Methylprednisolone 120 mg IV for 3 days under the cover of Amoxicillin/clavulanic acid. The treatment showed instant results as the fever subsided after 24H and the breast erythema and pain disappeared (Figure 3).

Steroid treatment was continued with prednisolone 60 mg/24H with daily dressing changes with paraffin-based gauze and wound washing with sterile saline which showed a good response, so we decided to discharge the patient five days after the diagnosis of PG was made, while continuing the treatment on an outpatient base with weekly visits and daily dressing changes by our home health care nurses.

**Figure 4.**
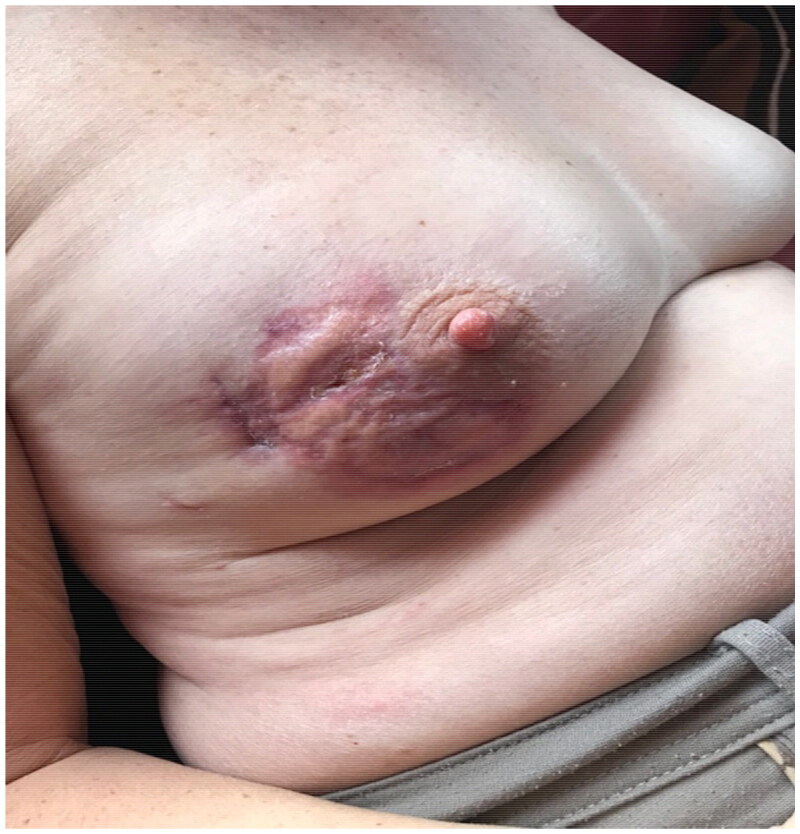
(POD45) The lesions healed completely approximately 1 month after the start of treatment and daily dressing changes.

**Figure 5.**
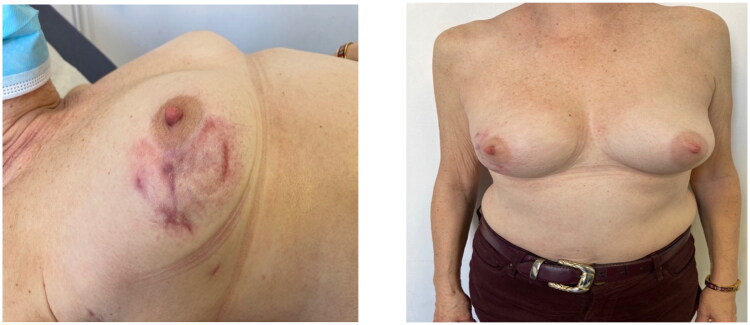
(POD90) 3 months after the surgery, complete recovery with minimal sequels due to the early diagnosis.

Eventually, the patient showed complete wound healing 1 month after the start of the treatment with a very acceptable aesthetic result within 3 months (Figure 4, 5) and her initial BCS margins were negative with no lymph node involvement so no further surgery or radiotherapy was required as hormonotherapy was sufficient for a hormonal positive HER negative tumor.

A maintenance therapy was left in place with progressive tapering of prednisolone for 6 months to avoid recurrence.

## Discussion

In 1930, Brunsting described the disorder PG [7] as an inflammatory neutrophilic dermatosis typically characterized by necrotizing ulceration and is believed to involve autoimmune dysfunction. Five clinical variants are currently recognized: classic, bullous, pustular, vegetative, and peristomal types [1].

Pathergy which is minor trauma leading to exaggerated skin injury is often associated with PG and is quite confusing for physicians in the setting of the post-surgical wound where the clinical features of PG can often be identical to simple post-operative wound infection [2].

It is very important for plastic surgeons to know how to clinically suspect PG of the breast in order to avoid excessive debridement that would only worsen the original ulceration and provide no benefits for patients. In certain cases, the time from presentation to diagnosis could take several years [8], so a high level of suspicion should be in order wherever there is breast tenderness, redness a few days after surgery with no response to antibiotics and usually negative wound cultures, with rapidly evolving ulcers sparing of the NAC.

PSPG of the breast is still considered rare as there have been less than 100 cases reported in the literature [4,9,10], Tuffaha et al. in their review reported the median time after surgery until the initial presentation of PG symptoms was 6 days, with fever present in (55%) and leukocytosis in (43%). wound cultures were found to be negative in (80%) of the cases reported. NAC was spared by the wounds in (89%)[11].

It is understandable that many surgeons are reluctant to start steroids without a confirmed diagnosis which is almost always the case in PSPG. However, a lower threshold should be in order when diagnostic criteria are fulfilled such as the criteria proposed by Delphi consensus including one major criterion (criteria) (biopsy of the ulcer edge demonstrating a neutrophilic infiltrate) and eight other minor criteria (exclusion of infection, pathergy, underlying systemic diseases, and cribriform scars at healed ulcer sites) [12] or the criteria proposed by Su et al. [13] shown in Table 1, where diagnosis requires both major criteria and at least two minor criteria.

## Risk factors

Many authors have addressed the relationship between PG and underlying systemic disease, most commonly inflammatory bowel disease, but also rheumatologic and hematological disorders such as leukemia, lymphoma, myelofibrosis, monoclonal gammopathy, and polycythemia vera [14,15].

As well multiple case reports have emerged with patients presenting with PG after solid malignancies mostly involving the breast whether it was after oncological resection or reconstructive surgery [16,17].

In their systemic review EHRL et al. [4] reported underlying associated malignancies, especially breast cancer, were noted in 32 cases (37%) and autoimmune conditions in 15 cases (17%). Of the 15 patients, four patients had a history of hematological disorders such as monoclonal gammopathy.

Similarly, ZUO et al. reported in their review of 220 cases of PSPG-associated risk factors such as a personal history of confirmed or suspected PG or in first-degree family members, suggesting a possible genetic predisposition [18].

## Treatment

Multiple treatment protocols have been described in the literature, First-line therapies include systemic corticosteroids and Cyclosporine A alone or in combination. In addition to Corticosteroid-sparing agents such as tacrolimus, methotrexate, azathioprine, infliximab, or adalimumab, topical therapy also has a role in early cases.

The initial dose, application form, schedule of steroid tapering, and steroid derivatives used were very variable. Empiric treatments include oral prednisone at 1 mg/kg/day, [1,18] intravenous methylprednisolone of 1–2 mg/kg/day [8,9,14] or Cyclosporine A of 5 mg/kg/day, Pulsed doses of methylprednisolone (1 g/day for five days) are an alternative regimen.

In addition to medical treatment dressing changes play a major role in healing, as more trauma would lead to worsening of the present ulcers due to pathergy, so surgical debridement should be kept to a minimum in the acute settings. The gold standard of care for these patients involves the use of NPWT especially in larger wounds, a modality that has been proven to be safe and effective in decreasing the healing time while preparing the local wound bed for skin grafting or secondary healing safely when combined with systemic immunosuppressors as mentioned by Almeida IR, et al. in their systemic review [19]. In our reported case the early recognition and hence smaller ulceration and faster treatment response permitted a simpler wound care protocol without the need for NPWT.

To prevent PG recurrence, Zakhireh et al. strongly recommended prophylactic steroid or immuno-modulator therapy, such as cyclosporine or azathioprine, during surgery to be tapered gradually over 6 months [20].

## Conclusion

PSPG remains an elusive diagnosis of exclusion that results in high morbidity which further adds to the importance of early recognition of clinical signs and symptoms associated with it, in order to ensure early medical treatment and decrease the need for unnecessary interventions in the acute phase and delayed reconstructive procedures later on.

## Consent form

All authors consent to submit the manuscript. Patient consent was acquired.
